# Case of Meningitis in a Neonate Caused by an Extended-Spectrum-Beta-Lactamase-Producing Strain of Hypervirulent *Klebsiella pneumoniae*

**DOI:** 10.3389/fmicb.2017.01576

**Published:** 2017-08-15

**Authors:** Khalit S. Khaertynov, Vladimir A. Anokhin, Yuri N. Davidyuk, Irina V. Nicolaeva, Svetlana V. Khalioullina, Dina R. Semyenova, Evgeny Y. Alatyrev, Natalia N. Skvortsova, Levon G. Abrahamyan

**Affiliations:** ^1^Department of Children Infectious Diseases, Kazan State Medical University Kazan, Russia; ^2^Institute of Fundamental Medicine and Biology, Kazan Federal University Kazan, Russia; ^3^Intensive Care Unit No 2, Republican Clinical Infectious Diseases Hospital Kazan, Russia; ^4^Laboratory for Animal Molecular Virology, Research Group on Infectious Diseases in Production Animals and Swine and Poultry Infectious Diseases Research Center, Faculty of Veterinary Medicine, Université de Montréal, Saint-Hyacinthe QC, Canada

**Keywords:** *Klebsiella pneumoniae*, extended-spectrum β-lactamases, hypervirulent, meningitis, neonate

## Abstract

*Klebsiella pneumoniae* is one of the most important infectious agents among neonates. This pathogen has a potential to develop an increased antimicrobial resistance and virulence. The classic non-virulent strain of *K. pneumoniae*, producing an extended-spectrum beta-lactamases (ESBL), is associated with nosocomial infection mainly in preterm neonates. Hypervirulent *K. pneumoniae* strains are associated with invasive infection among previously healthy ambulatory patients, and most of them exhibit antimicrobial susceptibility. During the last few years, several cases of diseases caused by hypervirulent *K. pneumoniae* producing ESBL have been registered in different geographical regions of the world. However, reports of such cases in neonates are rare. Here, we reported that this pathogen can cause pyogenic meningitis in full-term neonate with poor prognosis. A previously healthy, full-term, 12-day-old neonate was admitted to the infectious diseases hospital with suspected meningitis. The clinical symptoms included loss of appetite, irritability, fever, seizures, and a bulging anterior fontanelle. The analysis of the cerebrospinal fluid confirmed the diagnosis of meningitis. Blood and cerebrospinal fluid cultures were positive for *K. pneumoniae*, producing ESBL. *K. pneumoniae* isolates were resistant to aminopenicillins, 3rd generation cephalosporins but were sensitive to imipenem and meropenem. The “string test” was positive. The study of the virulence factors of *K. pneumoniae* by PCR revealed the presence of the *rmpA* gene. A combination of *K. pneumoniae* virulence and drug resistance complicated by cerebral oedema led to the death of the neonate. We concluded that both the risk of developing severe forms of infection and the outcome of the disease due to *K. pneumonia* are associated with the phenotypic features of the pathogen such as its antibiotic susceptibility and virulence factors. Emergence of the ESBL-producing strain of hypervirulent *K. pneumoniae* could represent a new serious threat to public health, suggesting an urgent need to enhance clinical awareness and epidemiological surveillance.

## Introduction

### Subject

Here, we report a case of neonatal meningitis caused by an ESBL-producing hypervirulent *Klebsiella pneumoniae* strain in a 12-day-old, male neonate. A 40-week-gestation male, with a birth weight of 3400 grams was born by cesarean delivery to a 24-year-old woman. The infant’s Apgar scores were 8 and 9 at delivery, and he was discharged on the 5th day after delivery. The mother gave no history of infections before delivery and no complications during pregnancy. Blood and cerebrospinal fluid cultures (CSF) of the infant patient were positive for *K. pneumoniae*.

The aim of this study was to determine the antibiotic susceptibility and virulence factors of *K. pneumoniae* isolated from a neonate with purulent meningitis. We investigated the antibiotic susceptibility of *K. pneumoniae*, the ability of the microorganism for production of extended-spectrum β-lactamases (ESBL), and its virulent factors: the *rmpA* gene and the hypermucoviscosity phenotype identified by a positive string test.

The Institutional Review Board of the Republican Clinical Infectious Diseases Hospital approved this study and written informed consent was obtained from a subject’s parents, according to the guidelines approved under this protocol (Federal Law “Protection of Health Right of Citizens of Russian Federation” N323- FL, 11.21.2011).

### Bacterial Isolates

One milliliter of blood sample was collected by a sterile syringe and mixed with 20 ml of Brain-Heart Infusion broth (Conda Pronadisa, Spain). This mixture was incubated at 37°C for 7 days, streaked onto the surface of blood agar and MacConkey agar (Oxoid, United Kingdom), and incubated at 37°C for 24 h (Collee et al., 1996). In order to isolate *K. pneumoniae* from CSF, a chocolate agar and blood medium were used (Oxoid, United Kingdom). Bacterial isolates were identified according to morphological and biochemical tests (Gram stain, capsule stain, motility test, indole production test, urease production test, Methyl Red test, Voges–Proskauer test) (MacFaddin, 2000) and confirmed by matrix-assisted laser desorption-ionization time-of-flight mass spectrometry (Microflex, Bruker Daltonics, Bremen, Germany).

#### Gram Stain

Bacterial isolates were resuspended in normal saline for Gram stain. Smear was prepared on a glass slide, air dried and fixed by gentle heating. The slide was flooded with crystal violet for 1 min. The stain was washed off with excess of tap water. Gram’s iodine was poured over the slide for 1 min. The slide was washed and destained with ethyl alcohol. Finally, counter staining was done with safranin for 30 s. Slides were washed again, dried and examined under the microscope.

#### For Capsule Staining

India Ink method was used. We placed a single drop of India ink on a clean microscope slide. Then, we removed some colonies from culture plate with a flamed loop and mixed them in the drop of India ink. Then, we placed the end of another clean microscope slide at an angle on the original slide containing the microorganisms. Next, we spread drop out so that it formed a thin film. After 5 min, we saturated the slide with crystal violet for 1 min and carefully rinsed it with water. The slide was air-dried for 5 min. Then, the slide was examined under the microscope using an oil immersion objective lens and looked for purple cells surrounded by a clear halo on a dark background.

#### Motility Test

Colonies of isolated microorganisms from an 18–24 h culture were inoculated into the medium by stabbing the center of the medium (HiMedia, India) to a depth of 1,5 cm. The inoculated medium was incubated at 37°C for 18 h. A positive motility test was indicated by a diffuse zone of growth flaring from the line of inoculation.

#### For the Indole Production Test

Conventional tube method was used. Colonies of microorganism were inoculated in tryptophan broth and were incubated at 37°C for 24 h in ambient air. Then, 0,5 ml of Kovac’s reagent was added to the broth culture. The test was considered positive if a pink colored ring appeared after the addition of reagent. Negative test is indicated if no color change occurred after the addition of reagent.

#### Urease Production Test

Colonies of isolated microorganism from an overnight Brain-Heart Infusion broth were streaked onto the surface of a urea agar slant. The tube of medium was incubated at 37°C in ambient air for 7 days. The test was positive if a pink color appeared.

#### Methyl Red and Voges–Proskauer Tests

Colonies of isolated microorganism were inoculated into Methyl Red/Voges–Proskauer broth tube. The tube of medium was incubated at 37°C for 24 h. After incubation, we removed two – 1 mL aliquots and placed them into two small tubes: one tube was for the methyl red test and the other for the Voges–Proskauer test. For the *Methyl Rose test*, we added five drops of methyl red to one tube. A red color at the surface was considered a positive result. For the *Voges–Proskauer test*, we added 0.6mL of 5% alpha naphthol, followed by 0.2 mL of 40% potassium hydroxide and shook the tube gently. A positive test was represented by the development of a red color 15 min after the addition of the reagents.

### Antibiotic Susceptibility Testing

The antibiotic susceptibility of *K. pneumoniae* isolates was determined by the Kirby-Bauer disk diffusion method according to Clinical Laboratory Standards Institute guidelines (CLSI) (Patel et al., 2015). Suspension of *K. pneumoniae* isolate was spread by sterile glass rods on the surface of *Mueller Hinton agar* (Oxoid, United Kingdom). Then antibiotic disks (Bio-Rad, France) were placed onto the surface of the inoculated *Mueller Hinton agar* plate. The plate was then incubated at 37°C for 18 h. Antimicrobial susceptibility was determined by measuring the diameter of the inhibition zone according to CLSI (2015). All antibiotics used for this test are listed in **Table 1**.

**Table 1:** The susceptibility of *K. pneumoniae* to antibiotics.

Antibiotic	Concentration (mg)	Values of diameter of inhibition zones (mm)	Susceptibility
Ampicillin	25	0	R
Ampicillin/clavulanate	30	0	R
Ceftriaxone	30	0	R
Ceftazidime	30	0	R
Cefotaxime	30	0	R
Ciprofloxacin	5	13	I
Co-trimoxazole	25	0	R
Imipenem	10	25	S
Meropenem	10	24	S
Amikacin	30	16	I
Gentamicin	10	0	R

*S, susceptible; I, intermediate; R, resistant.*

### Test for Production of Extended-Spectrum β-Lactamases (ESBL)

The *K. pneumoniae* isolates were tested for ESBL by using the double-disk method according CLSI (Patel et al., 2015).

Amoxicillin/clavulanate disks were placed in the center of *Mueller Hinton* agar plate (Oxoid, United Kingdom). The disk of ceftazidime and cefotaxime were placed at the distance of 20 mm from the amoxicillin/clavulanic acid disk. The plates were incubated aerobically at 37°C for 18 h before the zone size recorded. A positive result was indicated when the inhibition zones around any of the cephalosporin disks was augmented in the direction of the disk containing clavulanic acid.

### Hypermucoviscosity Testing

Single colonies, cultured on Brain Heart infusion agar plates (Conda Pronadisa, Spain), were obtained and tested for their ability to form viscous strings. The hypermucoviscosit*y* was defined by the formation of viscous strings > 5 mm length (Yu et al., 2007; Shon et al., 2013).

### DNA Extraction

Some colonies from the surface of MacConkey agar were suspended in 50 μl of sterile water. Total DNA was extracted from suspended cells using an extraction kit (“Litech,” Russia) according to the manufacturer’s recommendations.

### PCR Detection of Virulence-Associated Genes

DNA samples were analyzed using polymerase chain reaction (PCR) with primer pair for the *rmpA* (5′-ACGACTTTCAAGAGAAATGA-3′ forward and 5′-CATAGATGTCATAATCACAC-3′ reverse). Amplification was performed using C1000 Thermo Cycler (“Bio-Rad Laboratories,” United States) applying the following program: (1) DNA denaturation at 94°C, 3 min; (2) 35 cycles at 94°C, 30 s; 45°C, 30 s; 72°C, 35 s; (3) final extension at 72°C, 5 min; (4) reaction termination at 4°C. The amplicons were separated in 1% agarose gel and purified by using GeneJET Gel Extraction Kit (Thermo Scientific, United States) according to the manufacturer’s recommendations. The PCR-products were sequenced using the 3730 DNA Analyzer (Life Technologies, United States) to confirm the presence of the *rmpA* gene.

### Case

**FIGURE 1:**
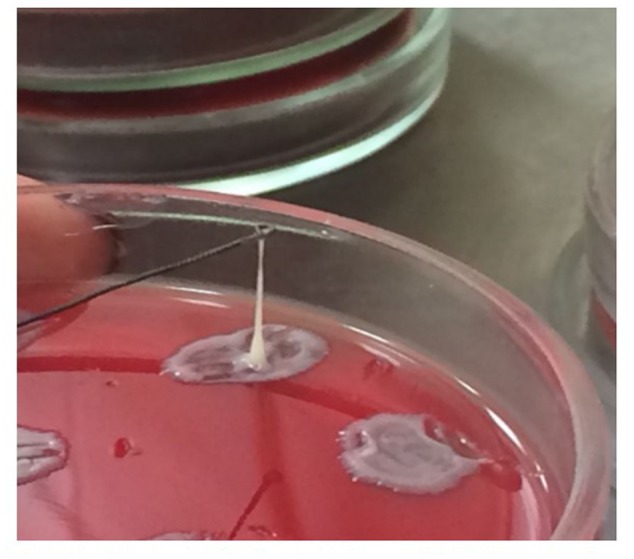
Hypermucoviscosity phenotype of *Klebsiella pneumoniae.* When the colonies were touched with a loop and the loop lifted vertically from the surface of the blood agar plate, the mucoid isolates adhered to the loop.

A full-term, 12-day-old, male neonate was admitted to the infectious diseases hospital with suspected meningitis on the 3rd day of illness. During the first 2 days, irritability and a loss of appetite were observed. On admission day, the infant had a temperature of 39°C and seizures. He looked noticeably ill and sleepy. On physical examination, his anterior fontanelle was bulging. The skin was pale, without rash. Chest, abdomen, and heart examinations did not show any abnormalities. The heart rate was 156 beats per minute, respiratory rate – 40 per minute. The liver and spleen were not enlarged. Chest X-ray was normal. The initial blood analysis revealed increased C-reactive protein (100 mg/L), presepsin (2932 pg/mL) and procalcitonin (more than 10 ng/mL). The initial complete blood cell count (CBC) analysis did not reveal any changes. CBC showed a erythrocytes count of 5,4 × 10^12^/L, a white blood cell (WBC) count of 6,4 × 10^9^/L, with 22% segmented neutrophils, 62% lymphocytes, 15% monocytes, 1% eosinophils, and 235 × 10^9^/L platelets. A lumbar puncture was performed: CSF was turbid, physico-chemical examination showed 21 000 WBC/mm^3^ with 90% neutrophils, 10% lymphocytes. CSF protein was 290 mg/dL, glucose – 0,3 mmol/L. Serum glucose was 8 mmol/L. The latex antigen test was negative for *Haemophilus influenzae B, Neisseria meningitides, Escherichia coli*, and *Streptococcus* group B. The cranial ultrasonography was performed and demonstrated thickening of the ventricular walls. The course of disease was complicated by the development of cerebral edema. The Pediatric Glasgow Coma Scale Score was 5. Changes in WBCs were found only on the 3rd day after hospitalization (**Table 2**). WBC examination showed 28.3 × 10^9^/L leukocytes with 77% neutrophils. The duration of leukocytosis and neutrophilia were 15 and 23 days, respectively. From the 2nd day of the hospitalization, the platelet count dropped to 25 × 10^9^/L. The duration of thrombocytopenia was 8 days. Blood and CSFs were positive for *K. pneumoniae* producing ESBL. Colonies of bacteria isolated on media were gray, mucoid, with diameters up to 2–4 mm, gram-negative, contained a thick capsule, and were non-motile (motility test was negative). Colonies of bacteria were positive for urease and *Voges–Proskauer* tests, and were itive negative for indole and Methyl Red tests. CSF culture for other bacteria was negative. The “string test” was positive (**Figure 1**). The study of the virulence factors by PCR revealed the presence of the *rmpA* (regulator of the mucoid phenotype) gene. The treatment of the patient included comprehensive antibiotics (ampicillin, amikacin, meropenem, cefoperazone), dexamethasone, IgM-enriched intravenous immunoglobulin, infusion therapy and mechanical ventilation. On admission day, a neonate was started on ampicillin (200 mg/kg/day) and amikacin (10 mg/kg/day). Both antibiotics a neonate received within 3 days, but did not show any clinical improvement. Following the isolation of *K. pneumoniae* (after 3 days), meropenem (120mg/kg/day) was administered, which a neonate received for 15 days. From the 19th day after hospitalization until the death, the patient received cefoperazone (100mg/kg/day). Despite the therapy, the patient died on the 35th day of the disease. The post-mortem examination revealed purulent meningoencephalitis, ventriculitis with the outcome of total cerebral leukomalacia, scattered pulmonary atelectasis, bilateral pneumonia, and the depletion of the thymus and spleen.

**Table 2:** Complete blood cells counts during hospitalization.

Days of hospitalization	1	3	8	15	23
Erythrocytes, × 10^12^/L	5,4	3,3	3,4	3,5	3,6
Hb, g/dl	142	133	135	137	132
WBC, × 10^9^/L	6,4	28,3	59,3	25,1	6,4
Banded, %	0	1	8	3	1
Segmented, %	22	76	62	70	74
Eosinophils, %	1	2	4	1	0
Lymphocytes, %	62	18	21	24	20
Monocytes, %	15	3	5	2	5
Platelets, × 10^9^/L	235	31	105	410	238

*Hb, hemoglobin; WBC, white blood cells.*

## Background

*Klebsiella pneumoniae* is one of the leading causes of hospital-acquired infection and neonatal sepsis (Janda and Abbott, 2006; Jones, 2010; Camacho-Gonzalez et al., 2013). The risk of severe bacterial infections such as sepsis and meningitis in neonates is associated mainly with neonatal factors: prematurity, low birth weight, and immaturity of innate and adaptive immunity (Cuenca et al., 2013; Cortese et al., 2016). Additionally, pathogenic features of the *K. pneumoniae* such as virulence and antibiotic resistance can define the course and outcome of the infection (Al-Hasan et al., 2011; Jacob et al., 2013). *K. pneumoniae* is an opportunistic pathogen often resistant to multiple antibiotics. During the last few decades, ESBL positive *K. pneumoniae* isolates have been recovered worldwide, especially in intensive care units (ICU) (Canton et al., 2008; Gelband et al., 2015). ESBLs can inactivate all penicillins and cephalosporins, including 3rd generation cephalosporins (Gelband et al., 2015). In neonates, *K. pneumoniae* median resistance to ampicillin was 94% and cephalosporins 84% in Asia; 100% and 50% in Africa (Le Doare et al., 2015). The prevalence of ESBL-producing strains of *K. pneumoniae* in the United States is 23%, in some countries of Europe up to 85–100% (Gelband et al., 2015). These microorganisms can cause outbreaks of neonatal sepsis in hospitals and neonatal ICU (Haller et al., 2015; Khaertynov et al., 2016). Virulence factors of *K. pneumoniae* play an important role in the development of infection. The factors that are implicated in the virulence of *K. pneumoniae* strains include the capsular polysaccharide, lipopolysaccharide, fimbrial adhesins, and siderophores (Broberg et al., 2014; Li et al., 2014). In the mid-1980s and 1990s, reports from Taiwan described cases of disease caused by hypervirulent strains of *K. pneumoniae* (hv-KP) (Liu et al., 1986; Cheng et al., 1991; Wang et al., 1998). A combination of clinical and bacterial phenotypic features of the hv-KP distinguishes it from the “classic” *K. pneumoniae* strains (Shon et al., 2013). One of these features is its ability to cause severe invasive infection (liver abscesses, meningitis, endophthalmitis) in previously healthy ambulatory patients. The second distinctive characteristic is the hypermucoviscous phenotype, which results in mucoid colonies on agar plates. This phenotype is defined by a positive “string test.” A positive string test is indicated by a microbiological inoculation loop able to generate a viscous string > 5 mm the length by stretching bacterial colonies on an agar plate (Yu et al., 2007; Shon et al., 2013). The hypermucoviscous phenotype of *K. pneumoniae* is associated with the presence of *rmpA* and *rmpA2* genes (Decré et al., 2011; Bialek-Davenet et al., 2014). The hypervirulent strains of *K. pneumoniae* currently spread throughout the world (Decré et al., 2011; Bialek-Davenet et al., 2014). Until recently, it was believed that the classic, non-virulent strains of *K. pneumoniae* producing ESBL and the hv-KP strains evolved separately, and have been considered as independent of each other. However, in 2014 an hv-KP able to produce ESBL (Zhang et al., 2015) was found in China, and in 2015 a clinical case caused by hv-KP producing ESBL was reported in France (Surgers et al., 2016). It is likely the frequency of these cases will grow worldwide. These cases were mainly observed in adult patients. The reports of such cases in neonates are extremely rare (Shankar et al., 2016).

## Discussion

Neonatal infection can occur in term infants because of birth risk factors and exposure to pathogens in the community as well. Neonatal sepsis and meningitis are more common in preterm newborns with low gestational age and low birthweight (Kavuncuoğlu et al., 2013; Samuelsson et al., 2014). *K. pneumoniae* infection is a typical nosocomial infection in neonates with the combination of these factors. However, in the case reported here, the disease occurred in a full-term baby. The mother of the baby did not have clinical signs of infectious disease. The neonatal meningitis ensues when pathogenic virulence factors overcome host defense mechanisms. For instance, the presence of a capsule at cell surface protects *K. pneumoniae* from opsonization and phagocytosis by macrophages and neutrophils (Li et al., 2014). On the other hand, capsular polysaccharide of *K. pneumoniae* suppress the early inflammatory response by inhibition of IL-8 expression through the inhibition of TLR4 signaling (Li et al., 2014). Thus, the pathogenic potential of the microorganism plays an important role in the disease outcome. In the reported case, the *K. pneumoniae* isolates produced ESBL and were absolutely resistant to aminopenicillins, 3rd generation cephalosporins and gentamicin, with diameters of inhibition zones of 0 mm for each of them. *K. pneumoniae* isolates were sensitive only to imipenem and meropenem with diameters of inhibition zones of 25 and 24 mm, respectively. Intermediate sensitivity was observed for amikacin and co-trimoxazole with diameters of inhibition zones of 16 mm and 13 mm, respectively. Additionally, these isolates were hypervirulent (i.e., positive ”string test” and presence of *rmpA*). In this case, neonatal meningitis was caused by ESBL-producing strain of hv-KP. An unfavorable outcome has been associated with the inefficiency and late onset of antibacterial therapy, as well as the formation of a severe inflammatory reaction from the membranes and brain matter. Bacterial meningitis in neonates is an independent risk factor for mortality: mortality rates of meningitis caused by *K. pneumoniae* reaching 17.1% (Watson et al., 2003). We noted the absence of changes in WBC on admission day despite the massive pyogenic process in the cerebrospinal fluid. Given the fact of bacteremia, this unusual situation with the redistribution of neutrophils in newborn fluids cannot be explained only by the biological characteristics of the microorganism. Likely, the nature of the immune response to a severe invasive infection in a newborn patient takes place. The primary reaction of the newborn child in the tissues of the central nervous system is associated with the redistribution of neutrophil chemoattractants: with an increase in their concentration in the cerebrospinal fluid, their level in the blood should be reduced. We regret that we were unable to confirm this assumption. For example, simultaneous assessment of interleukin-8 in the blood and cerebrospinal fluid could have provided confirmation. However, we do not rule out that such changes are associated with specific biological characteristics of the microorganism. Meningitis caused by hypervirulent *K. pneumoniae* is associated with potential mortality. Emergence of hypervirulent strains of *K. pneumoniae* producing EXBL can represent a major challenge for patient treatment. In our study, the most sensitive antibiotics to hypervirulent ESBL-producing *K. pneumoniae* strains were meropenem and imipenem. In cases of neonatal sepsis or neonatal meningitis caused by hypervirulent strains of *K. pneumoniae* producing EXBL, these two carbapenems should be considered as first-line therapy and should be administered as soon as possible.

## Concluding Remarks

The risk of developing severe forms of infection due to *K. pneumonia* and the outcome of the disease are associated not only with neonatal factors (low gestational age, very low birth weight), but also with the features of the microorganism: its antibiotic susceptibility and virulence. ESBL-producing strain of hypervirulent *K. pneumoniae* causes invasive infection (pyogenic meningitis) in full-term neonate with poor prognosis.

## Author Contributions

KK design of the study and wrote the paper. Discussed the results and implications, wrote the manuscript. VA design of the study and wrote the paper. YD DNA extraction of *K. pneumoniae* strains with subsequent genotyping by PCR method to determine virulence factors. IN design of the study. SK wrote the paper. DS collection of clinical data. EA collection and interpretation of clinical data. NS isolation of *K. pneumoniae* colonies, determination of their antibiotic resistance and ability to produce of extended spectrum β-lactamase. LA wrote the paper. Discussed the results and implications, wrote the manuscript.

## Conflict of Interest Statement

The authors declare that the research was conducted in the absence of any commercial or financial relationships that could be construed as a potential conflict of interest.
